# Characterizing the Effects of Calcium and Prebiotic Fiber on Human Gut Microbiota Composition and Function Using a Randomized Crossover Design—A Feasibility Study

**DOI:** 10.3390/nu13061937

**Published:** 2021-06-04

**Authors:** Lara S. Yoon, Karin B. Michels

**Affiliations:** 1 Department of Epidemiology, Fielding School of Public Health, University of California, Los Angeles, CA 90095, USA; lyoon6@ucla.edu; 2 Institute for Prevention and Cancer Epidemiology, Faculty of Medicine and Medical Center, University of Freiburg, 79110 Freiburg, Germany

**Keywords:** dietary intervention, crossover, gut microbiota, short chain fatty acid, prebiotic, calcium

## Abstract

Consumption of prebiotic inulin has been found to increase calcium absorption, which may protect against gut diseases such as colorectal cancer. This dietary relation may be modulated by compositional changes in the gut microbiota; however, no human study has addressed this hypothesis. We determined the feasibility of a randomized crossover trial to evaluate the effect of three interventions (combined calcium and inulin supplementation, calcium supplementation alone, and inulin supplementation alone) on the intestinal microbiota composition and function. We conducted a 16-week pilot study in 12 healthy adults who consumed the three interventions in a random sequence. Participants provided fecal and blood samples before and after each intervention. Each intervention period lasted four weeks and was flanked by one-week washout periods. 16S ribosomal RNA sequencing and quantification of short chain fatty acids (SCFA) was determined in fecal samples. Systemic lipopolysaccharide binding protein (LBP) was quantified in serum. Of the 12 individuals assigned to an intervention sequence, seven completed the study. Reasons for dropout included time (*n* = 3), gastrointestinal discomfort (*n* = 1), and moving (*n* = 1). Overall, participants reported positive attitudes towards the protocol (*n* = 9) but were unsatisfied by the practicalities of supplement consumption (44%) and experienced digestive discomfort (56%). We found no appreciable differences in microbial composition, SCFA concentration, nor LBP concentrations when comparing intervention periods. In conclusion, an intervention study using a randomized crossover design with calcium and a prebiotic fiber is feasible. Improvements of our study design include using a lower dose prebiotic fiber supplement and a larger sample size.

## 1. Introduction

The gastrointestinal (GI) tract has long been recognized as a central axis of human health [1]. This centralized role has been further enhanced through the expansive research over the last two decades on the microbial inhabitants of the GI tract, the gut microbiota. Notably, the bacterial composition of the gut microbiota has been associated with and causally implicated in the development of many diseases (asthma, autism, anxiety, type I and II diabetes, obesity, inflammatory bowel disease) [2]. Moreover, the gut microbiota exhibits a variety of metabolic activities, producing a wealth of excretory products reported to stimulate the differentiation of immune cells, regulate host cell metabolism, and even modify psychosocial behaviors [3,4].

Research highlighting the role of diet and dietary supplements in modulating the composition and function of the gut microbiota is extensive. Calcium and inulin represent dietary components with known effects on the gut microbiota [5,6,7]. Inulin is a prebiotic fermentable fiber that is indigestible by human cells and promotes the growth of beneficial gut microbes [8]. It serves as a key nutrient for gut microbes that convert inulin and other soluble plant fibers into short-chain fatty acids (SCFAs) via fermentation [8]. Calcium is a nutrient commonly found in dairy products and leafy vegetables that is inversely associated with body weight. Though the mechanisms remain unclear, hypotheses suggest that calcium exerts influence on health outcomes through modulation of the gut microbiota [9]. Consumption of inulin has also reported to increase calcium absorption, particularly in the large intestine [10,11]. While inulin has been studied extensively in relation to the gut microbiota, and calcium to a lesser extent, few studies have examined their combined effect. This highlights an understudied avenue of microbiota research, by which fermentable fibers and calcium might instigate gut microbial compositional variations that could protect against negative health outcomes. 

An interventional approach to combine calcium and prebiotic fiber has great potential to provide the public with modifiable lifestyle factors to support overall gut health. However, there are challenges in designing, implementing, and analyzing human dietary interventional studies which assess gut microbiota-related outcomes, including confounding introduced by inter-individual variation in lifestyle or diet, sample size, and compliance [12,13]. Moreover, there is a wealth of potential markers available for evaluation of gut microbial outcomes, including abundance of specific bacterial taxa, function, and metabolic capacity in various sample types (feces, blood, urine). To this end, large intervention trials on the human gut microbiome benefit from pilot and feasibility studies [14].

Here, we discuss results from a 16-week randomized crossover dietary intervention feasibility study comprised of 12 healthy adult men and women. The primary goal of this study was to evaluate the feasibility of the crossover design protocol, the compliance of the participants, and the tolerability of the interventions. The crossover study design, in which participants are randomly assigned to a sequence of consecutive interventions interspersed with a washout period, is efficient and reduces participant variation, as each participant serves as their own control [15]. However, this design is typically longer in length than a parallel-intervention study and thus requires feasibility testing. A secondary goal was to obtain preliminary data on the impact of calcium and prebiotic inulin fiber on gut microbiota composition and function.

## 2. Materials and Methods

### 2.1. Participant Eligibility and Recruitment

Healthy adults aged 18–35 years were recruited via informational flyers at the University of California, Los Angeles in Westwood, CA, USA. Twenty-seven potential participants were screened via telephone for eligibility. Twelve men and women aged 18–35 years, currently not smoking, or consuming any nutritional/dietary supplements, with no familial or personal history of colorectal cancers or other intestinal disorders (e.g., inflammatory bowel disease), and having not taken oral/intravenous antibiotics within two months of the study start date were enrolled in this study and randomized to an intervention sequence. Participants were asked to refrain from more than two alcoholic drinks per week, maintain their typical diet, and avoid consumption of nutritional or probiotic supplements during the study period. 

### 2.2. Experimental Design and Dietary Interventions

The study was conducted at the University of California, Los Angeles (UCLA) between January and June 2018. All recruitment and study protocols were approved by the Institutional Review Board at UCLA (IRB#17-001129). All participants gave signed informed consent prior to beginning the study.

**Figure 1:**
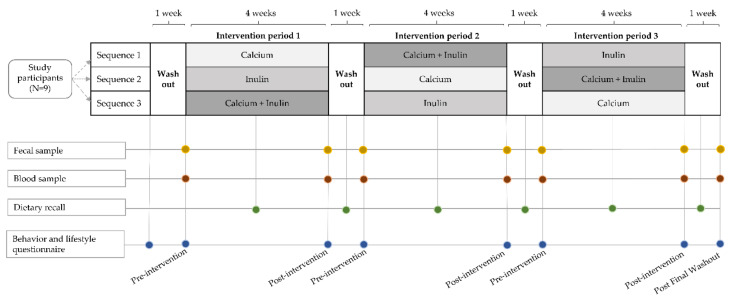
Illustration of the study design, a 3 × 3 randomized crossover intervention pilot study comparing the effects of three interventions, calcium supplementation, inulin supplementation, and combined calcium and inulin supplementation, on gut microbiome parameters. Participants (*n* = 9) were randomized into a sequence of three four-week intervention periods interspaced by one-week washout periods. Fecal and blood samples were collected at the start and end of each intervention period and after the last washout period. Dietary recalls were collected at the middle of each study period. Behavior and lifestyle questionnaires were collected at the study start and end, and before and after each intervention period.

The study was conducted in a randomized crossover design spanning 16 weeks with three four-week intervention periods and a one-week washout period between interventions (Figure 1). Of note, in a crossover trial, every participant receives each intervention; however, the sequence of the interventions differs for every participant. Using this design, each participant serves as their own control or comparison. Intervention periods are interspersed with wash-out periods, hence comparison can be made between the different interventions as well as between washout period and intervention periods. Blood and fecal samples were collected at the start and end of each study intervention period. During the three intervention periods, subjects consumed daily powder supplements of calcium alone (‘calcium’), inulin alone (‘inulin’), or a combination of the two (‘combination’). Participants were randomized into an intervention sequence by the study coordinator using simple randomization in Microsoft Excel. Analyses were performed without knowledge of the intervention sequence or period. After randomization, participants began a one-week initial washout period. This was considered the start of the study at which time participants were required to adhere to all dietary restrictions.

Throughout the study, visits to supply subjects with supplements and collect fecal and blood samples and questionnaire data took place at the Clinical Translational Research Center (CTRC) at the University of California, Los Angeles. At each study visit, a trained phlebotomist collected blood for serum isolation, and the study coordinator received the fecal sample and provided the participants with supplements for the next intervention period. Participants were asked to complete general health questionnaires and 24-h dietary recalls six times throughout the study. 

A final sample collection of feces and blood occurred after the last washout period (post final washout), marking the end of the study period. In total, seven fecal and blood samples were collected from each participant throughout the study.

Calcium carbonate powder and inulin powder were purchased in bulk via NOW foods and provided to participants at the start of each intervention period. Participants were asked to self-measure and consume 2 g of calcium powder, 15 g of inulin, or a combination of 2 g of calcium and 15 g of inulin each day during the relevant intervention period. Participants were instructed to mix each supplement in a glass of juice or other liquid to consume once per day.

### 2.3. Questionnaires

Participants were asked to complete a baseline questionnaire prior to beginning the study. The baseline questionnaire included information about ethnic/racial background, age, weight, height, current medications, supplement use, drug and alcohol use, dietary habits, and general health. Participants were also asked to self-report how often (days per week) they consumed foods high in calcium, fiber, or sugar, as well as their red-meat consumption.

At the end of each intervention and washout period, participants were again asked about their general health, including frequency of bowel movements and stool mass via questionnaire. Six times throughout the study, during each intervention and washout period, participant dietary intake data (24-h recalls) were collected and assessed using the Automated Self-Administered 24-h (ASA24) Dietary Assessment Tool, version24–2018, developed by the National Cancer Institute (Bethesda, Rockville, MD, USA) to ensure compliance with the dietary restrictions of the study.

Participants also completed an exit questionnaire which assessed attitudes towards eight different components of the study in a Likert-type manner following completion of the study. In the exit questionnaire, participants rated their attitudes towards the following topics: ease of following dietary guidelines, ease of stool collection, ease of blood collection, ease of powder consumption, discomfort following powder consumption, clarity of study instruction, and organization of study. Each component was evaluated using a quantitative scale from one to five, with five being the most positive response, three being neutral, and one being the most negative.

### 2.4. Sample Collection

Fecal samples were collected by participants at-home using the EasySampler^®^ Stool Collection Kit (ALPCO) at baseline and at the end of each intervention and washout period. Detailed instructions with images and text were provided for standardized collection of the at-home stool sample. Participants were instructed to collect fecal samples in the 24 h prior to their study visit and to store fecal samples at 4 °C until that time. All fecal samples were aliquoted into 2 mL aliquots for SCFA and 16S ribosomal RNA (rRNA) gene sequencing within 24 h of collection and stored at −80 °C until further processing.

Twenty mL of blood was collected in red-topped tubes for serum isolation from each participant at baseline and the end of each intervention and washout period by a trained phlebotomist. Immediately after collection, blood samples were left to clot at room temperature for 15–20 min. Serum was then isolated by centrifuging the samples at 1500× *g* for 10 min at 4 °C. Serum was aliquoted into 2 mL cryovials and stored at −80 °C until further processing.

### 2.5. 16S rRNA Sequencing

The fecal microbiota was analyzed via 16S rRNA gene sequencing. Fecal samples were submitted to the University of California, Los Angeles Microbiome Core Lab for DNA extraction, library preparation, purification, and quantification, and Illumina HiSeq paired-end sequencing of the V4 region of the 16S gene using a previously described method [16,17]. In brief, total bacterial DNA was extracted using the ZymoBIOMICS DNA kit (cat no. D4300) with bead beating. The V4 region of the 16S gene was amplified and barcoded using 515f/806r primers (forward-barcoded: GTGYCAGCMGCCGCGGTAA; reverse-barcoded: GGACTACNVGGGTWTCTAAT) then 250 × 2 bp sequencing was performed on an Illumina HiSeq 2500. Raw data were processed using DADA2 scripts in R platform and operational taxonomic units (OTUs) were identified by closed reference picking against the Silva database [18]. Taxonomic classification occurred prior to any statistical analysis.

Standard preprocessing was used to exclude undefined or ambiguous taxa and taxa below a prevalence threshold of 2% of total samples [19]. No samples were excluded. The final OTU feature table comprised 4,378,557 total reads (mean per sample = 72,976, range 5894 to 142,480), 1110 taxa, and 60 samples. 

### 2.6. Short-Chain Fatty Acid, Lipopolysaccharide Binding Protein, and Zonulin Analyseis

Gas-chromatography (GC) quadrupole mass spectrometry (MS) for analysis of fecal SCFAs was conducted at the University of California, Davis West Coast Metabolomics Center following a previously described protocol [20]. The following SCFAs were quantified: acetic acid, butyric acid, propionic acid, formic acid, and iso-valeric acid. The GC-MS analysis of samples was done using Agilent technologies (Agilent 7890 GC/Agilent 5977 MS). 

Lipopolysaccharide binding protein (LBP) analysis was performed by the CER Laboratory at Boston Children’s Hospital. LBP was measured by a quantitative sandwich enzyme immunoassay technique from Hycult Biotech (Plymouth Meeting, PA, USA). A monoclonal antibody specific for LBP was pre-coated onto a microtiter plate. Samples, standards, and controls were added to the plate where the LBP binds; after an incubation, unbound materials were washed from the plate. A second LBP antibody labeled with biotin was added, incubated and again washed to remove unbound compounds. Then, a streptavidin-peroxidase conjugate was added, the streptavidin binds to the biotin and again the plate is washed. A substrate was added to react to the peroxidase, resulting in a color generated that is proportional to the amount of LBP present in the sample. The assay has a sensitivity of 1.0 ng/mL and possesses a day-to-day variability of less than 10% over a wide range of LBP concentrations. 

Concentrations of zonulin in the serum samples were measured by a competitive enzyme immunoassay from ALPCO Diagnostics (Salem, NH, USA) and performed by the CER Laboratory. The assay possesses a sensitivity of 0.140 ng/mL and day-to-day variabilities at zonulin concentrations of 41.13 and 46.15 ng/mL of 7.7% and 8.3%, respectively.

### 2.7. Statistical Analyses

Descriptive statistics were used to characterize participants in the study. Continuous data were presented as mean and standard deviation (SD) or median when appropriate. Categorical data were summarized by number and frequency (*n*, %). 

Alpha diversity was computed using the Shannon diversity index [21]. Between-group differences in the Shannon index were estimated using analysis of variance (ANOVA). Beta diversity was calculated on filtered and normalized data using the Bray–Curtis dissimilarity metric [22]. Principal coordinate analysis (PCoA) using Bray–Curtis dissimilarity was used to visualize the largest sources of variation in the data. Permutational analysis of variance (PERMANOVA) was performed to test for composition differences between intervention groups [23]. Diversity indices and plots were produced using the phyloseq and vegan packages for R [24,25]. 

Multivariate Association with Linear Models (MaAsLin) was used to analyze differential abundance of individual bacterial taxa between study interventions. Post-intervention bacterial taxa abundance was used as the outcome measure; model coefficients compare each intervention to the others. All MaAsLin models are unadjusted under the assumption that randomization to crossover sequence was successful. Models additionally include subject-specific random effects to account for non-independence between samples as a result of the longitudinal study design. The minimum abundance of taxa included in the analysis was set to 0.0001 and the minimum prevalence was set to 0.2. This indicates that the taxa needed to be present in at least 20% of the samples at an abundance of at least 0.01%. Relative abundances were transformed using Trimmed Mean of M. We report all unadjusted *p*-values less than 0.05 and accompanying Benjamini–Hochberg false discovery rate–adjusted *q*-values. Linear modeling was done using the MaAsLin2 package in R [26].

ANOVA and Tukey’s Test were used to compare the effect of the three study interventions on post-intervention SCFA and LBP concentrations. Statistical tests were performed at the 0.05 level of significance. MaAsLin models were also used to examine associations between specific microbial taxa and circulating LBP concentrations, measured post-intervention. SCFA- and LBP- analyses were performed in R (version 4.0.2).

To evaluate the possibility of carryover effect from one intervention period to the next, we compared baseline alpha diversity, SCFA concentration, and LBP concentration to the pre-intervention periods and the post final washout period using paired *t*-tests (Supplementary Materials).

## 3. Results

### 3.1. Baseline Cohort Characteristics

**Figure 2:**
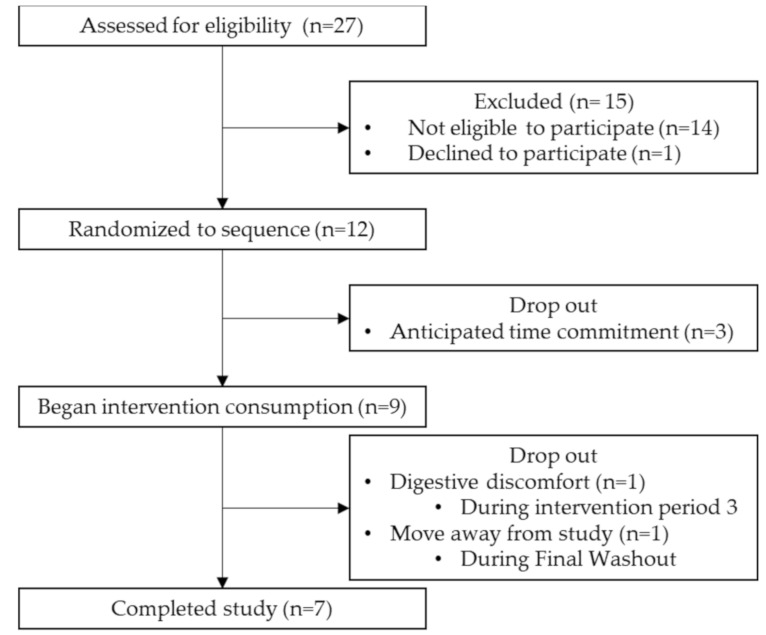
Study flow chart for the process of selecting eligible participants. The period from Randomized to sequence to Completed study comprised 16 weeks. N is in weeks.

We screened 27 potential participants recruited through flyer distribution. A total of 12 participants, aged 18–35 years, were eligible to participate, enrolled in the study and randomized into an intervention sequence (Figure 2). Of the 12 participants that initially enrolled in the study, nine completed the baseline questionnaire and began the first intervention, and seven completed the full study. Reasons for dropout during the first intervention period were due to the time commitment (*n* = 3). Dropouts that occurred towards the end of the study (after periods two and three, respectively) were due to digestive discomfort (*n* = 1) and moving away from the study location (*n* = 1).

The majority of participants in this study was female (77.8%) and identified as non-Hispanic White (55.6%) (Table 1). The mean age of the cohort participants was 26 years. The average body mass index (BMI) of the participants was 21.9 kg/m^2^; all participants had a BMI in the normal/healthy range (18.5 to 24.9 kg/m^2^). At baseline, all of participants rated their health as excellent (22.2%) or good (77.8%). Similarly, all participants rated the healthiness of their diet as excellent (33.3%) or good (66.7%). On average, participants self-reported consuming foods high in calcium 4.7 days per week; foods with fiber, 3.9 days per week; foods with added sugar, 2.4 days per week; and red meat, 2.4 days per week. The mean number of bowel movements per week was 8.7. Participants reported engaging in some form of physical activity 3.2 days per week. 

### 3.2. Feasibility and Post-Study Assessments 

Throughout the study, participants were asked to complete questionnaires regarding their digestive health status, noting any concerns related to the interventions or sample collections (Table 2). A greater number of participants reported experiencing abdominal or gastrointestinal discomfort during the combination inulin and calcium intervention (55.6%) than during the inulin-alone intervention (12.5%) or the calcium-alone intervention (11.1%). More participants also reported an increase in bowel movements, including more diarrhea, and an increase in the number of days experiencing gastrointestinal discomfort during intake of the combination intervention.

Nine participants completed the exit questionnaire (Table 3). Participants reported positive attitudes towards study components related to easiness of following dietary guidelines and ease of powder consumption during the sample collection, as well as clarity of instructions. Participants reported feeling neutral about the level of digestive comfort following powder consumptions. Overall, participants were very interested in the study and felt the study was very organized. 

### 3.3. Microbiota Compositional Changes Associated with Calcium and Inulin Consumption

**Figure 3:**
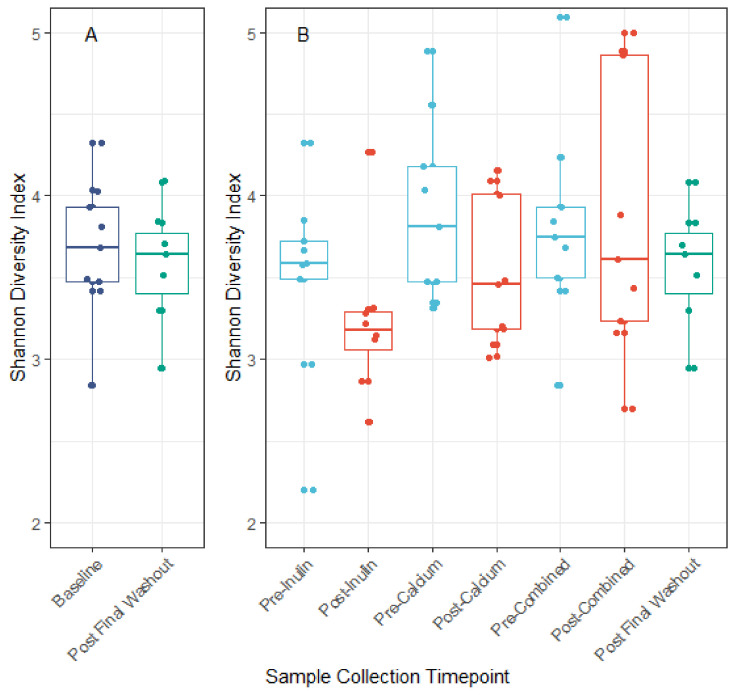
Alpha diversity measured by the Shannon diversity index. Panel A: Comparison of Shannon diversity index at baseline and post final washout. Panel B: Comparison of Shannon diversity index before and after each intervention period. No significant differences in alpha diversity across interventions were noted when comparing the baseline to post final washout (*p* = 0.68) nor when comparing across all sample timepoints (ANOVA *p* = 0.21).

**Figure 4:**
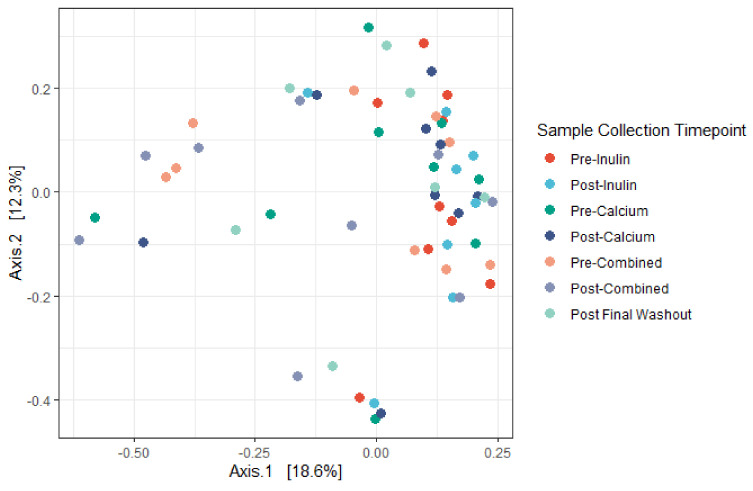
Principal coordinates analysis (PCoA) derived from Bray–Curtis dissimilarity among all samples. No significant differences in centroid (Adonis, *p* = 0.42) nor dispersion (PERMANOVA, *p* = 0.192) were noted. Axes correspond to principle coordinates displaying the maximum amount of variance (%) in the dataset. (*n* = 9 for all sample collection timepoints except for the final washout, *n* = 7, and post-inulin, *n* = 8).

We identified minor negative shifts in median alpha diversity when comparing the pre-inulin to post-inulin fecal samples (Figure 3). No obvious shifts were detected for other interventions, though variance for the sample timepoints was large. Furthermore, as apparent by the lack of significant clustering on the PCoA plot, the phylogenetic relation between subjects is not driven by supplement consumption (Figure 4). Examination of the plot according to subject ID suggests that some clustering may be driven by the genetic background and/or environmental factors specific to each subject (Figure S1). No significant differences in alpha diversity were noted for pairwise comparisons of baseline to the pre-intervention (post-washout) periods (Figure S2).

**Figure 5:**
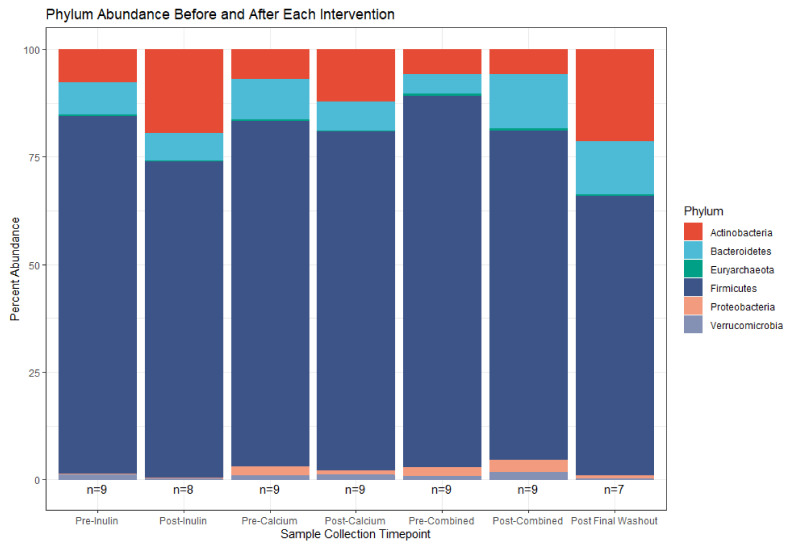
Relative phylum abundance in the top 100 OTUs before and after each supplement. (*n* = 9 for all sample collections except for the final washout, *n* = 7, and post-inulin, *n* = 8).

Despite the lack of global shifts in the gut microbiota community, analysis of bacterial relative abundances between supplements highlights some distinct compositional shifts. This is particularly apparent in some of the less abundant bacterial phyla (e.g., Proteobacteria and Verrucomicrobia, Figure 5). Consumption of calcium alone decreased the abundance of Proteobacteria while consumption of inulin alone decreased the abundance of Verrucomicrobia. Following consumption of the combined supplement, the abundances of these two phyla were increased and, relative to the remaining sample collection time points, there was an outgrowth of Bacteroidetes. The post-calcium and post-inulin intervention samples shifted abundance of members of the Bacteroidetes phylum in the negative direction compared to their pre-intervention counterparts; the opposite effect was seen for the combined intervention.

MaAsLin was used to identify differentially abundant taxa associated with supplement consumption. OTUs identified with unadjusted *p*-value ≤ 0.05 are specified in Table 4. Of these OTUs, four were differentially abundant when comparing the calcium intervention to the inulin intervention. An additional 12 OTUs were differentially abundant when comparing the combined intervention to the Inulin intervention. None of the OTUs were differentially abundant when adjusting for multiple testing (*q*-values > 0.05). 

### 3.4. Calcium and Inulin Consumption Are Not Associated with Shifts in Fecal SCFA Concentrations

**Figure 6:**
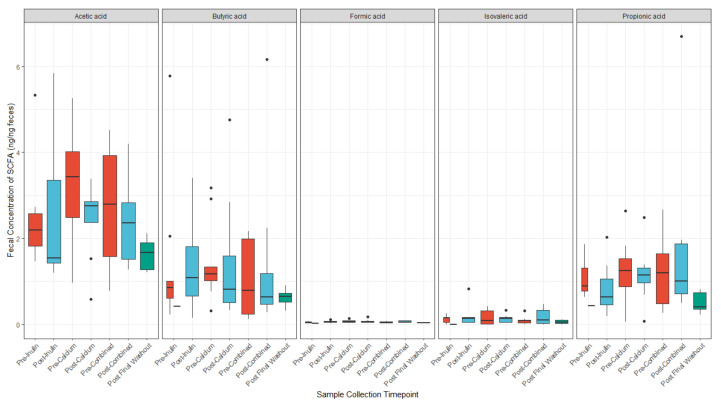
SCFA concentrations (ng/ng feces) before and after each supplement. (*n* = 9 for all sample collections except for the final washout, *n* = 7, post-inulin, *n* = 8, and post-calcium, *n* = 8).

We compared concentrations of the fecal SCFAs, acetic acid, butyric acid, formic acid, isovaleric acid, and propionic acid, at each sample collection timepoint (Figure 6). Isovaleric acid was below the limit of detection for five of the samples. On average, there were higher concentrations of acetic acid among participants in the study at all time points, followed by butyric and propionic acid. However, there were no major differences in any of the SCFA concentrations after consumption of any of the supplements. There were minor shifts in concentrations of acetic acid, butyric acid, formic acid, and propionic acid from baseline to pre-intervention and post-final washout samples (Figure S3). 

### 3.5. The Role of Calcium and Inulin in Modulating Systemic LBP and Zonulin Concentrations 

**Figure 7:**
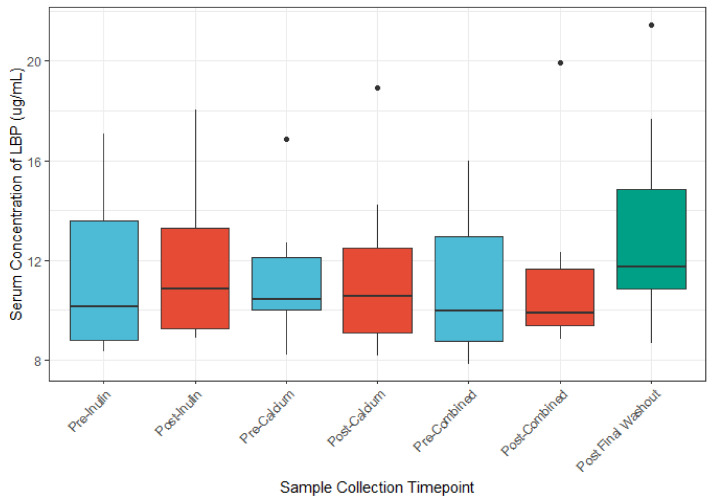
LBP concentrations (μg/mL) before and after each intervention. (*n* = 9 for all sample collections except for the final washout, *n* = 7, post-inulin, *n* = 8).

We identified no appreciable differences in serum LBP concentrations between the before or after supplement comparisons (Figure 7). Multivariable analysis of the association between LBP concentration and OTU abundance identified nine OTUs whose abundances were associated with LBP concentrations (unadjusted *p* ≤ 0.05; Table 5). However, after application of the Benjamini–Hochberg method for reduction in false discovery rate, only one OTU remained differentially abundant. No differences in LBP were noted for pairwise comparison of baseline fecal samples to Pre-Intervention (post-washout) periods (Figure S4). There were no major differences in zonulin concentration across sample timepoints (Figure S5).

## 4. Discussion

In this small-scale pilot study, we demonstrated the feasibility of a 16-week dietary intervention study using a randomized crossover design to examine the effect of the intake of calcium and a prebiotic supplement on the composition and function of the gut microbiome. 

A key component of this pilot study was the assessment of the feasibility of our study design. Several recent studies have evaluated the impact of dietary interventions on gut microbial outcomes using the crossover design; most studies were done in a 2 × 2 design, with two intervention groups and two intervention periods ranging from 1 week to ≥12 weeks and corresponding washout periods of 1 to ≥12 weeks [27,28,29,30,31,32]. The four-week intervention and one-week washout periods in our study were chosen to reflect the current evidence for the temporal effects of inulin-type fructans and to minimize duration-related dropout for potential study participants [33]. 

Key challenges in long-term dietary intervention trials are attrition and compliance associated with ability to maintain the dietary intervention of interest [34]. The length of our study was a limiting factor with regard to feasibility. Three participants withdrew after giving consent and going through randomization but before beginning the initial washout period due to the time commitment the study would require. The study spanned 16 weeks and participants were required to adhere to dietary restrictions and sample collection schedules for the duration of this time period. Combined with the participant that withdrew due to unwanted effects of the intervention, this suggests that ~33% of enrolled participants can be anticipated to withdraw before the study is completed. Other crossover intervention studies evaluating the microbiome have reported similar rates of attrition; the study participation period for these studies ranged from 13 weeks to 12 months [27,29,35]. While it is possible that the baseline questionnaire was too cumbersome for three study participants, studies have suggested that a shorter questionnaire has minimal consequences with improving retention [36]. To mitigate such high rates of early dropout in a larger intervention study we propose improved communication of study requirements including the duration and burden of this study, particularly during the screening phase. Other successful retention strategies in larger trials have included multi-method communication—including email, phone, and social media messaging—as well as increased monetary incentives [37]. 

We requested feedback from our participants throughout the study as a measure of feasibility. Participants enrolled in our study reported little to no concerns with regard to the number of blood draws and fecal collections. However, one participant withdrew for gastrointestinal discomfort presumably related to supplement consumption. Other prebiotic intervention studies have reported similar dropout due to gastrointestinal symptoms [38]. Consumption of a supplementary inulin is reported to increase stool frequency and decrease stool transit time [39]. Depending on the normal frequency of bowel movements and diet of the participant, varied effects on the gut health of the participants and possible loss to follow-up should be anticipated in future studies. Several participants reported a neutral or negative attitudes towards consumption of the supplement powders. Our protocol required daily intake of 15 g of inulin powder for two of the study periods, and several participants noted the difficulty in mixing the supplement powder into their daily beverages. While our study did not use an unusual amount of inulin (other studies have reported 5–30 g per day), a larger study may benefit from use of a prebiotic fiber supplementation with a lower daily dose requirement [40]. For example, xylooligosaccharide (XOS) is an efficacious prebiotic plant fiber found to be associated with fecal microbial abundance and has a much lower effective dose (1.4–2.8 g/day) in intervention studies [41,42]. Overall, this suggests that XOS may be a more attractive option compared to inulin for study participants with respect to supplement burden. 

The randomized crossover design enabled us to compare the effects of three interventions in the same participant, thereby minimizing the influence of the substantial inter-individual variation in the core microbiome. In general, the crossover study design can be more efficient than a parallel-treatment design, requiring fewer subjects [15]. However, the crossover design is susceptible to carryover effects if the washout period is not sufficiently long to allow for return to baseline. We found limited evidence to support a carryover effect in our study. Minor shifts in concentrations of four of the SCFAs were found when comparing the baseline fecal sample to those take after the washout periods. The recommended duration for a washout period in a dietary intervention study with gut microbiota-associated outcomes is unclear. Other intervention studies have utilized washout periods ranging from 6 days to more than three weeks, but evidence to support a consistent temporal return to baseline remains inconclusive [43]. One study reported a return to baseline with as little as a six-day washout period [44]. Given that most participants found the 16-week length to be acceptable, we would consider a longer washout period in a larger trial to mitigate any carryover effect. 

Overall, taxonomic composition of the gut microbial community in our study is consistent with gut microbiomes reported by others. For instance, members of the Firmicutes phylum represent the majority of inferred organisms in our study across all sample time points, followed by the Bacteroidetes and Actinobacteria phyla. Studies of the gut microbiome which use 16S rRNA gene sequencing-based methods report similar dominance by members of the Firmicutes and Bacteroidetes phyla [45]. That we found no major changes in the composition of the gut microbiota throughout the study period may be due to our limited sample size. We hypothesized that supplementing the diet of healthy adults with a daily combination of calcium and inulin powder or a daily calcium powder would shift the composition of the gut microbiome when compared to inulin alone. Many studies have consistently demonstrated changes in the abundance of intestinal microbial taxa (most commonly Bifidobacterium) following intervention with inulin prebiotic supplements among adult populations [46]. Calcium has also been reported to modify the composition of the gut microbiome in limited studies [47,48]. Moreover, we found no shifts in overall species richness nor in community differentiation when comparing interventions. We did find a small shift in Shannon diversity following the inulin intervention. A similar decrease in alpha diversity was also found in a single-group dietary inulin-type fructan intervention study [6]. Long-term temporal analyses suggests that the gut microbiome is relatively stable [49,50]. Given that our study consisted of a healthy adult population, it is possible that our interventions were not substantial enough additions to the average diets to trigger changes in microbial diversity. 

Our analysis of fecal SCFA concentrations suggests that combined supplementation with inulin and calcium does not affect the metabolic function of the gut microbiota. Indeed, there was no difference in SCFA concentrations when comparing the combined calcium and fiber intervention to calcium or inulin alone. Supplementary analyses also found no changes in SCFA concentration following any of the dietary interventions. SCFAs are by-products of bacterial fermentation of carbohydrates, and human cells utilize SCFAs as energy sources [3,4]. Furthermore, these microbial metabolites are reported to influence the differentiation and activity of human immune cells [3,51]. The microbiota degrades prebiotic components and produces SCFAs as by-products of this degradation (23). Given the reported positive correlation between dietary fiber and SCFA concentration in many recent studies, the lack of significant effect of the inulin alone and combination interventions on SCFA concentration is surprising when compared to other studies [52]. However, that we also find no major compositional shifts in our study suggests that a null finding for comparisons SCFA concentration is not unusual. Both the duration of our interventions and the size of our cohort (*n* = 9) considerably limits our ability to confirm if these supplements may alter gut microbiota metabolic function.

Serum concentrations of LBP after each intervention period were not different from baseline, nor were they different when comparing across the post-intervention periods. We report a mean and median LBP concentration consistent with other reported levels of LBP in studies of younger adults [53,54]. Studies suggest that gut microbial composition may influences levels of circulating LBP through alteration of the intestinal barrier; as such, LBP levels have been hypothesized as markers for intestinal barrier function [55]. However, few human studies have identified gut microbial taxa associated with LBP concentrations. In a cross-sectional study of premenopausal women, members of Bacteroides were found at higher abundance among women with higher (>22 μg/mL) circulating levels of LBP, while *Christensenellaceae* bacteria were found in higher levels among women with low levels of LBP (<15 μg/mL) [56]. We report only one taxa associated with LBP concentration (*Eggerthella lenta*). A randomized controlled feeding study of 17 participants found a positive association of *E. lenta* with vegetable intake [57]. The available studies are limited by sample size. 

Because this was a feasibility study with a small sample size, we have offered cautious interpretation of our scientific results. Beyond the acknowledged limitations in sample size, another limitation of our study is the lack of effective control of intra- and inter-individual variation in the microbiota. We collected one baseline sample from the study participants and observed minor variations in microbiota composition and metabolic function in the samples collected prior to each intervention. We also assume that with the crossover design, there were no intra-person shifts in normal diet or lifestyle behaviors. Collectively, these data highlight the need for enhanced statistical modeling strategies to adjust for potential shifts in microbiota composition and function over time. Additionally, the transportability of our study may be limited. The study participants were recruited using flyers at the University of California, Los Angeles, and comprised of primarily students and research staff. The sample may thus be more educated, younger, and healthier than the general population. Effort to recruit from a broader population should be considered in a larger trial.

There are a number of strengths to our study. Importantly, this study allowed for assessment of recruitment and resulting sample characteristics, including retention rates, eligibility criteria, and timing. We were also able to evaluate the acceptability of the data collection process and suitability of the interventions for participants by collecting information on participant attitudes towards study procedures. A notable strength of this study is the crossover intervention study design, which allows for a more efficient study with a smaller sample size. Because of these within participant comparisons, the statistical power of the study is significantly enhanced. This is also the first study to evaluate the influence of a combined prebiotic intervention of inulin and calcium. Another strength of this study is the use of a multi-omics approach for evaluating the gut microbiome. The use of 16S rRNA sequencing in complement with SCFA and LBP analyses enables a more integrated assessment of composition and function of the gut microbiome.

## 5. Conclusions

In summary, we find that a randomized crossover design to evaluate the effects of combined fiber and calcium supplementation on the gut microbiome is feasible and would benefit from the implementation of additional strategies for participant retention, and application of modeling strategies to adjust for intraindividual variation in the microbiota.

## Figures and Tables

**Table 1:** Baseline characteristics of participants who began the randomized crossover dietary intervention study (*n* = 9).

Characteristic	*n*	%
Female	7	77.8
Age, years (mean, SD)	26.1	3.0
BMI, kg/m^2^ (mean, SD)	21.9	1.9
Weight, lbs (mean, SD)	131.4	20.5
Race		
Non-hispanic white	5	55.6
Hispanic/Latino	1	11.1
Asian	3	33.3
Diet		
Self-rated health of diet		
Excellent	3	33.3
Good	6	66.7
Fair	0	0
Poor	0	0
Self-reported weekly food consumption, days per week (mean, SD)		
Calcium	4.7	1.3
Fiber	3.9	0.9
Sugar	2.4	1.7
Red meat	2.4	1.9
Self-reported health		
Excellent	2	22.2
Good	7	77.8
Fair	0	0
Poor	0	0
Physical activity, days per week (mean, SD)	3.2	2.0
Bowel movements per week (mean, SD)	8.7	3.3

**Table 2:** Summary of recurring questionnaires throughout the study (*n* = 9).

	Inulin Intervention	Calcium Intervention	Combined Intervention
	*n* ^1^ (%)	*n* (%)	*n* (%)
Experienced abdominal pain or gastrointestinal comfort	1 (11.1)	1 (11.1)	5 (55.6)
Days/week experiencing GI discomfort			
0	6 (66.7)	7 (77.8)	4 (44.4)
≥1	2 (22.2)	2 (22.2)	5 (55.6)
Experienced changes in bowel movements	3 (33.3)	0 (0)	4 (44.4)
Bowel movements per week during the intervention period (mean [SD])	9.3 (3.6)	9.3 (3.9)	9.4 (5.6)

^1^*n* = 8 due to dropout.

**Table 3:** Summary of the exit questionnaire following completion of the study (*n* = 9).

Component	Mean ^1^ (SD)
Interest in topic	4.2 (0.9)
Ease of following dietary guidelines	4.4 (0.9)
Ease of stool sample collection	3.9 (1.3)
Ease of blood collection	4.6 (0.5)
Ease of powder consumption	2.9 (1.3)
Discomfort following powder consumption	3.1 (1)
Clarity of study instructions	5 (0)
Organization of study	5 (0)

^1^ Components were assessed on a scale of 1 to 5, with five being the most positive response, three being neutral, and one being the most negative. For example, for “interest in study topic”, a value of five corresponds to very interesting and a value of one corresponds to not-at-all interesting.

**Table 4:** Differentially abundant OTUs (unadjusted *p* ≤ 0.05) present in the gut microbiome post-intervention, comparing consumption of inulin alone (reference) to calcium alone or combined (*n* = 9).

OTU	Phylum	Genus; Species	Coef ^1^	SE	*p*-Value ^2^	*q*-Value ^3^
Comparison: Calcium vs. Inulin (reference)						
OTU125	Firmicutes	*Butyricicoccus;*	0.366	0.161	0.038	0.858
OTU20	Firmicutes	*Streptococcus;*	−0.582	0.260	0.040	0.858
OTU170	Firmicutes	*Christensenellaceae_R-7_group;*	0.375	0.171	0.044	0.858
OTU21	Firmicutes	*Anaerostipes; hadrus*	−0.360	0.167	0.048	0.858
Comparison: Combined vs. Inulin (reference)						
OTU89	Bacteroidetes	*Bacteroides;*	0.721	0.228	0.006	0.858
OTU300	Bacteroidetes	*Odoribacter; splanchnicus*	0.508	0.181	0.013	0.858
OTU6	Actinobacteria	*Bifidobacterium;*	−0.669	0.250	0.017	0.858
OTU158	Bacteroidetes	*Alistipes; putredinis*	0.609	0.232	0.019	0.858
OTU26	Firmicutes		0.611	0.239	0.022	0.858
OTU3	Firmicutes	*Anaerostipes; hadrus*	−0.307	0.129	0.031	0.858
OTU21	Firmicutes	*Anaerostipes; hadrus*	−0.397	0.167	0.031	0.858
OTU47	Firmicutes	*Roseburia; intestinalis*	−0.473	0.207	0.037	0.858
OTU189	Firmicutes	*Veillonella;*	0.812	0.356	0.037	0.858
OTU170	Firmicutes	*Christensenellaceae_R-7_group;*	0.371	0.171	0.047	0.858
OTU65	Firmicutes	*CAG-352;*	0.443	0.206	0.048	0.858
OTU39	Firmicutes		−0.202	0.095	0.050	0.858

^1^ A negative coefficient implies a decrease in the associated taxon. ^2^ *p*-values are unadjusted (nominal significance of this association). ^3^ *q*-values for significance produced using the Benjamini–Hochberg method for decreasing false discovery rate. Abbreviations: Coef, coefficient; SE, standard error.

**Table 5:** Associations between specific microbial taxa and circulating LBP concentration (*n* = 9).

Feature	Phylum	Genus; Species	Coef ^1^	SE	*p*-Value ^2^	*q*-Value ^3^
OTU105	Actinobacteria	*Eggerthella; lenta*	0.38	0.096	0.000	0.042
OTU8	Actinobacteria	*Bifidobacterium*	0.258	0.076	0.001	0.122
OTU258	Firmicutes	*Lachnoclostridium*	−0.293	0.095	0.003	0.200
OTU45	Actinobacteria	*Bifidobacterium; animalis*	0.341	0.117	0.005	0.200
OTU255	Actinobacteria	*Gordonibacter*	0.261	0.088	0.005	0.200
OTU273	Firmicutes	*Lachnospiraceae_UCG-004*	−0.199	0.076	0.017	0.551
OTU225	Firmicutes	*Ruminiclostridium_5*	−0.193	0.082	0.022	0.591
OTU108	Firmicutes	*Blautia*	0.169	0.076	0.029	0.696

^1^ A negative coefficient implies a decrease in the associated taxon. ^2^ *p*-values are unadjusted (nominal significance of this association). ^3^ *q*-values for significance produced using the Benjamini–Hochberg method for decreasing false discovery rate. Abbreviations: Coef, coefficient; SE, standard error.

## Data Availability

The data presented in this study are available on request from the corresponding author. The data are not publicly available due to privacy.

## Supplementary Materials

The following are available online at https://www.mdpi.com/article/10.3390/nu13061937/s1, Figure S1: PCoA assessing beta diversity of the microbiome according to the participants enrolled; Figure S2: Alpha diversity measured by the Shannon Diversity Index at Baseline, Pre-Intervention 2, Pre-Intervention 3, and Post-Final Washout; Figure S3: SCFA concentrations (ng/ng feces) measured at Baseline, Pre-Intervention 2, Pre-Intervention 3, and Post-Final Washout; Figure S4: LBP concentrations (μg/mL) measured at Baseline, Pre-Intervention 2, Pre-Intervention 3, and Post-Final Washout. Figure S5: Zonulin concentrations (ng/ml) measured in serum at each sample collection time point.
