# Supplementary material for: Characterizing the Effects of Calcium and Prebiotic Fiber on Human Gut Microbiota Composition and Function Using a Randomized Crossover Design—A Feasibility Study
Source: Nutrients. 2021 Jun 4;13(6):1937. doi: 10.3390/nu13061937 (PMC8227192; doi:10.3390/nu13061937)
Supplement: Supplementary file 1 [file nutrients-13-01937-s001.zip › nutrients-1139966-supplementary.pdf]

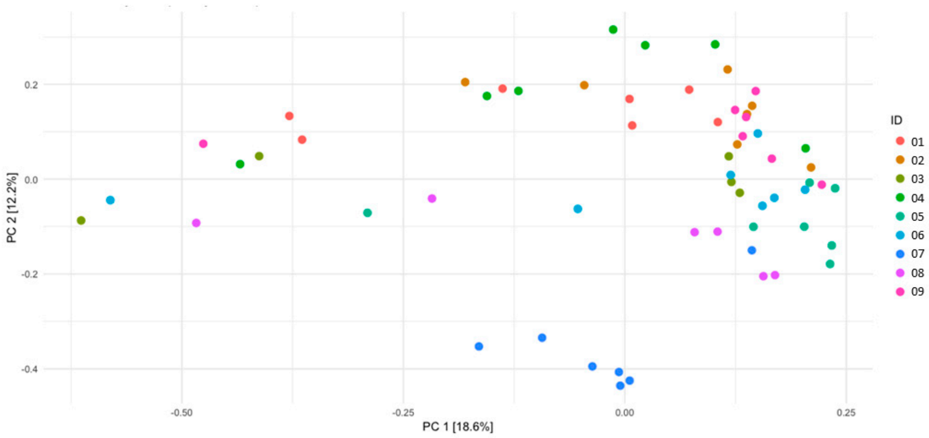

Figure S1. PCoA assessing beta diversity of the microbiome according to the participants (ID) enrolled. Axes correspond to principle coordinates displaying the maximum amount of variance (%) in the dataset. (n = 9 for all sample collections except for the final washout, n = 7, and inulin 2, n = 8).

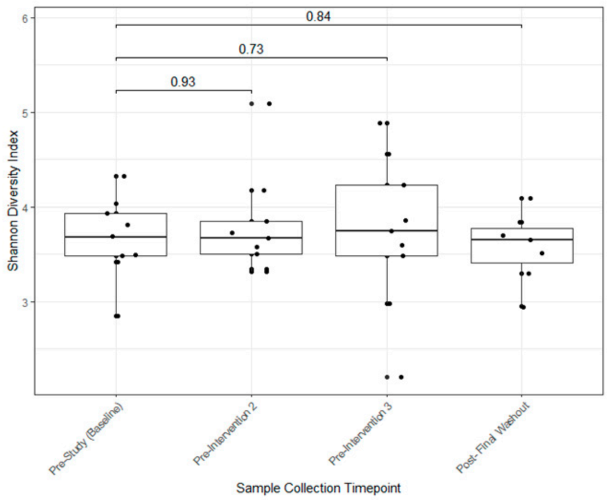

Figure S2. Alpha diversity measured by Shannon Diversity Index at Bsaseline, Pre-Intervertion 2, Pre-Interverntion 3, and Post-Final Washout. P-values are presented for paired t-test.

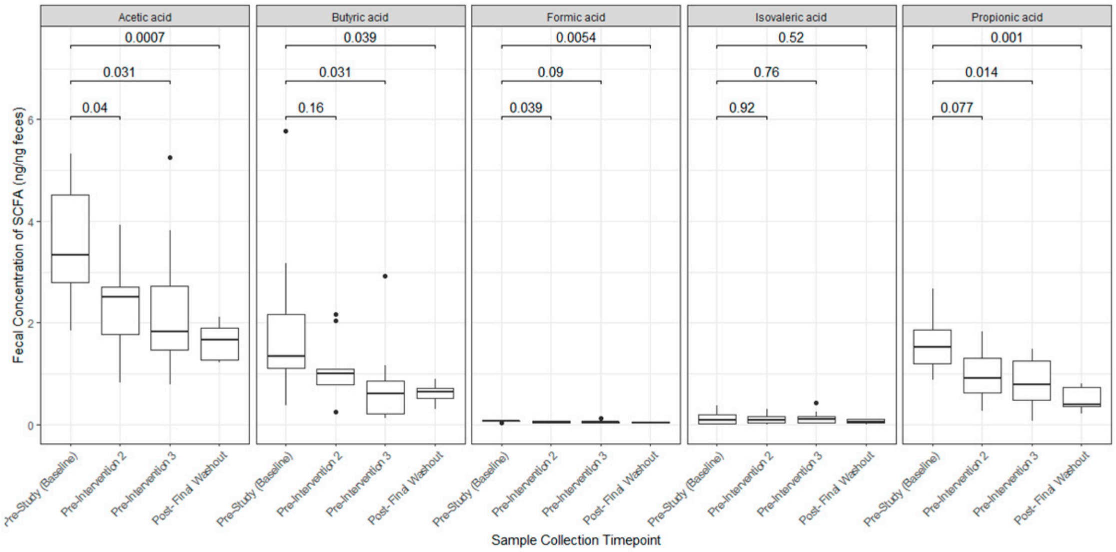

Figure S3. Short-Chain Fatty Acid (SCFA) concentrations (ng/ng feces) measured at Baseline, Pre-Intervention 2, Pre-Intervention 3, and Post-Final Washout. *P*-values are presented for paired *t*-test for the five SCFAs.

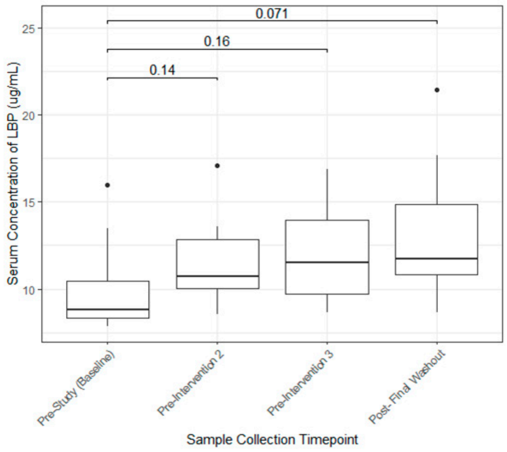

Figure S4. LBP concentrations (μg/mL) measured at Baseline, Pre-Intervention 2, Pre-Intervention 3, and Post-Final Washout. *P*-values are presented for paired *t*-test.

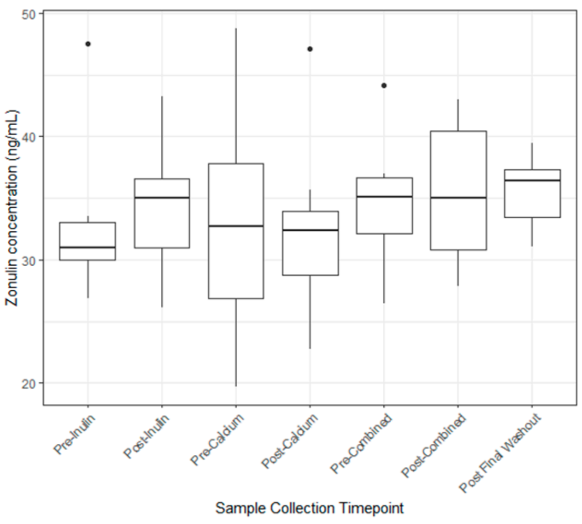

Figure S5. Zonulin concentrations (ng/mL) measured in serum at each sample collection time point. (*n* = 9 for all sample collections except for Post Final Washout, *n* = 7, and Post-Inulin, *n* = 8, and Post-Calcium, *n* = 8). No differences in concentration were noted (ANOVA, *p* = 0.93).
